# Whole genome capture of vector-borne pathogens from mixed DNA samples: a case study of *Borrelia burgdorferi*

**DOI:** 10.1186/s12864-015-1634-x

**Published:** 2015-06-06

**Authors:** Giovanna Carpi, Katharine S. Walter, Stephen J. Bent, Anne Gatewood Hoen, Maria Diuk-Wasser, Adalgisa Caccone

**Affiliations:** Department of Epidemiology of Microbial Diseases, Yale School of Public Health, 06520 New Haven, CT USA; Robinson Research Institute, University of Adelaide, 5005 Adelaide, SA Australia; The Geisel School of Medicine, Dartmouth College, 03755 Hanover, NH USA; Department of Ecology, Evolution and Environmental Biology, Columbia University, 10027 New York, NY USA; Department of Ecology and Evolutionary Biology, Yale University, 06520 New Haven, CT USA

**Keywords:** Hybrid capture, Whole-genome sequencing, SNPs, Tick-borne pathogens, Lyme disease

## Abstract

**Background:**

Rapid and accurate retrieval of whole genome sequences of human pathogens from disease vectors or animal reservoirs will enable fine-resolution studies of pathogen epidemiological and evolutionary dynamics. However, next generation sequencing technologies have not yet been fully harnessed for the study of vector-borne and zoonotic pathogens, due to the difficulty of obtaining high-quality pathogen sequence data directly from field specimens with a high ratio of host to pathogen DNA.

**Results:**

We addressed this challenge by using custom probes for multiplexed hybrid capture to enrich for and sequence 30 *Borrelia burgdorferi* genomes from field samples of its arthropod vector. Hybrid capture enabled sequencing of nearly the complete genome (~99.5 %) of the *Borrelia burgdorferi* pathogen with 132-fold coverage, and identification of up to 12,291 single nucleotide polymorphisms per genome.

**Conclusions:**

The proprosed culture-independent method enables efficient whole genome capture and sequencing of pathogens directly from arthropod vectors, thus making population genomic study of vector-borne and zoonotic infectious diseases economically feasible and scalable. Furthermore, given the similarities of invertebrate field specimens to other mixed DNA templates characterized by a high ratio of host to pathogen DNA, we discuss the potential applicabilty of hybrid capture for genomic study across diverse study systems.

**Electronic supplementary material:**

The online version of this article (doi:10.1186/s12864-015-1634-x) contains supplementary material, which is available to authorized users.

## Background

Next generation sequencing (NGS) technologies have transformed infectious disease molecular epidemiology, generating unprecedented amounts of high quality genomic data for a range of downstream applications. The rapidly declining cost, low error rate, and high throughput of existing second-generation platforms enables generation of whole-genome sequences (WGS) for inventory of standing pathogen genomic variation across space and time and development of powerful single nucleotide polymorphism (SNP) markers [1–4]. High density, genome-wide SNPs are critical for fine-resolution studies of pathogen transmission chains, population structure, phylogeography, phylogenomics, and for a range of genome-wide association studies (GWAS).

However, the power of NGS has not yet been fully harnessed for vector-borne and zoonotic disease (VBZ) systems, the majority of globally emerging infectious diseases [5]. Although NGS sequencing has provided valuable insight into the genomic diversity of easily culturable VBZ parasites such as *Plasmodium* spp. and *Trypanosoma* spp.[6–10], large-scale genomic studies of field-collected pathogens has been stymied by the difficulty of obtaining high-quality WGS data for pathogens more difficult to culture. This is due to the high ratio of host (the arthropod vector or the reservoir host) to pathogen DNA present in mixed DNA samples [11, 12]. The overwhelming presence of this exogenous DNA (which may also include commensal bacteria, endosymbionts, and environmental microbes) renders shotgun sequencing inefficient and cost-prohibitive. The development and availability of an efficient, and cost-effective method to retrieve WGS directly from field-collected VBZ samples will (1) enable study of pathogen demographic and evolutionary history, providing critical information to predict pathogen spread or monitor the success for epidemiological interventions, and (2) provide a wealth of genomic resources for association studies [13].

The agent of the most prevalent vector-borne disease in the US, the Lyme disease bacterium, *Borrelia burgdorferi* sensu stricto, exemplifies this common hurdle to genomic study for many VBZ systems. *B. burgdorferi’s* small genome (~1.5 Mb), is overwhelmed by the genome size of its black-legged tick vector, *Ixodes scapularis* (~2.1Gb) [14–17] and, in average field collected nymphal ticks, *B. burgdorferi* represents <0.01 % of the total DNA template. Pure *B. burgdorferi* cultures would be the ideal template for generation of genomic DNA libraries, enabling maximally efficient deep sequencing. However, culturing *B. burgdorferi* directly from its tick vector is labor intensive and culture success is strain-dependent, introducing significant bias [18]. Shotgun approaches for WGS of pathogens directly from field samples are inefficient due to the excess of contaminating host DNA present in the samples, which result in very low sequence coverage of the low copy number bacterial genomes present [11]. Recently, the use of selective whole genome amplification to amplify DNA from the target bacteria from mixed DNA samples has been proposed [19]. While this method enables enrichment of *B. burgdorferi* DNA from artificial mixtures of bacterial DNA, it was not validated for *B. burgdorferi* genome sequencing directly from field-collected ticks with spirochete burdens expected in natural infections. Furthermore, this method includes a PCR-based whole genome amplification step prior to sequencing, introducing several potential biases [20–22].

Despite the well documented increase in incidence of human Lyme disease over the last forty years [23], *B. burgdorferi* molecular epidemiology and population genetic studies have relied on analyses of single genes or multi-locus sequence typing based on eight housekeeping genes (MLST) [24–27]. Use of these coarse markers precludes fine-scale study of *B. burgdorferi* variation in the field and highlights the need for a population genomics approach for study of the epidemiology and biology of this important human pathogen.

The advent of hybrid capture (or hybrid enrichment) in synergy with NGS has overcome limitations related to low input and mixed DNA samples [21, 28, 29]. Using custom oligonucleotide arrays, hybrid capture harnesses the differential power of DNA hybridization to extract a genome of interest from a mixed template. This method was originally developed for human genome-wide association studies [28], where it was used to selectively re-sequence the human exome [29]. Hybrid capture does not require amplification prior to sequencing, thus circumventing the biases known to be introduced by whole genome amplification and similar methods [21, 22, 30]. Moreover, hybrid capture can enrich for divergent sequences, even when oligonucleotide arrays used to capture the targeted genome are derived from a single reference genome [31, 32]. Although this method has been successfully applied in a variety of systems [21, 29, 31–34] from ancient human DNA [35] to modern human clinical samples of *Plasmodium falciparum* [32], it has not yet been exploited for assessing pathogen genomic diversity directly from mixed DNA template specimens derived from arthropod vectors and/or animal reservoir hosts.

In this study, we demonstrate the efficiency of combining hybrid capture techniques and NGS to enrich for *B. burgdorferi* genomic DNA directly from field collected vector samples. Using this combined approach we *i*) evaluate the capture efficiency for *B. burgdorferi* genomes in different multiplexed capture strategies, *ii*) generate 30 *B. burgdorferi* WGS at high coverage, and *iii*) identify high quality genome-wide SNPs. This new efficient and cost-effective method can be readily applied to a variety of VBZ disease systems and enables us to take advantage of field collections for population genomic studies of emerging pathogens.

## Results

### Whole genome capture efficiency

#### Shotgun sequencing

Using one Hiseq2000 lane, we directly sequenced two whole tick DNA extracts (samples Sh834 and Sh1589, Table 1) with a *B. burgdorferi* load, measured by qPCR, within the expected range of field-collected nymphs (3721 and 1184 spirochete copies, respectively) [36]. This generated ~144 million reads (75 bp paired end reads) with a mean capture efficiency (the proportion of sequence reads mapping to our target of interest, the *B. burgdorferi* B31 reference genome) of <0.01 % (Table 1). The majority of remaining sequence reads (average: 66.49 %) mapped to the tick vector *I. scapularis* draft assembly. This represents an underestimate of tick-derived reads, as the ~66 % of the *I. scapularis* draft assembly consists of highly repetitive regions affecting the overall mapping efficiency [37–39].

**Table 1:** Mapping statistics. For each sample the first four columns list the sample names followed by the capture reaction (number of genomic libraries captured in multiplex), the total number of sequenced bases (Mb) prior to filtering, and the percentage PCR duplicates. The final five columns report the capture efficiency (percentage of reads that mapped to the B. burgdorferi B31 reference genome), the percentage of sequence covered by at least one generated sequence read and the fold coverage (the total number of unique sequence reads which map to each nucleotide in the reference B31 genome) of filtered data for the linear chromosome (911 Kb) and the longest linear plasmid, lp54 (53 Kb), respectively

Sample	Capture reaction	Sequenced bases (Mb)	% duplicate	Capture efficiency	% Chr covered	Coverage depth (Chr)	% lp54 covered	Coverage depth (lp54)
Bbcap1	20	34.22	42.63	0.44	99	11.71	98	12.77
Bbcap2_L1^a^	10	108.45	50.1	0.64	100	52.92	100	52.53
Bbcap2_L2^b^	4	237.68	70.49	0.61	100	109.52	100	113.51
Bbcap3	20	94.32	56.52	0.76	100	52.6	100	59.7
Bbcap4_L1	1	2066.79	92.57	0.10	100	140.83	98	194.75
Bbcap4_L2	4	188.60	61.81	0.56	100	76.92	100	94.68
Bbcap5	20	163.28	61.93	0.81	100	109.28	100	66.03
Bbcap6_L1	10	334.98	69.68	0.81	100	217.77	98	208.67
Bbcap6_L2	20	202.09	64.25	0.81	100	131.54	98	125.66
Bbcap7	20	75.67	55.01	0.65	100	39.08	98	34.93
Bbcap8	20	41.28	47.49	0.49	100	13.87	100	18.71
Bbcap9	10	221.18	67.38	0.76	99	134.32	98	148.14
Bbcap10	20	449.34	81.21	0.91	100	305.33	100	259
Bbcap12	20	185.23	67.57	0.77	100	103.49	98	91.28
Bbcap13	10	617.48	83.63	0.81	100	331.14	98	327.07
Bbcap14	20	140.75	62.89	0.70	100	76.99	98	86.69
Bbcap15	10	69.67	49.41	0.43	99	23.64	99	33.1
Bbcap16	20	119.57	54.98	0.72	99	71.59	99	71.73
Bbcap17	20	344.88	72.98	0.84	100	219.8	100	245.4
Bbcap19	10	53.31	41.46	0.53	100	19.73	100	25.17
Bbcap20	20	128.15	59.07	0.72	100	72.48	100	84.78
Bbcap21	20	135.54	60.87	0.71	99	72.32	98	79.68
Bbcap22	4	976.61	91.94	0.88	100	597.31	100	782.62
Bbcap23	20	159.50	63.51	0.64	99	77.6	98	105.44
Bbcap24	20	84.34	49.5	0.65	100	42.99	100	29.66
Bbcap25	20	61.60	50.95	0.50	99	25.13	98	28.68
Bbcap26	20	75.92	57.04	0.53	99	30.63	98	31.59
Bbcap27	20	73.49	60.97	0.59	100	33.45	100	35.05
Bbcap28	20	123.64	65.07	0.73	100	72.31	99	61.74
Bbcap29	10	255.63	72.17	0.86	100	167.46	100	178.06
Bbcap30_L1	10	343.08	70.18	0.83	100	225.25	99	183.62
Bbcap30_L2	4	825.19	88.1	0.79	100	520.17	100	438.71
Bbcap31_L1	10	292.77	65.76	0.85	100	198.23	100	136.51
Bbcap31_L2	20	232.33	66.08	0.85	100	156.58	100	109.34
Bbcap32	10	164.70	60.74	0.74	99	87.35	98	104.07
Sh834 ^c^	Shotgun	9954.53	19.92	6.00E–05	7.7	1.2	0.8	1.23
Sh1589	Shotgun	825.75	18.15	1.40E–04	27.1	1.389	38	1.5

^a^L1: Samples (indexed genomic libraries after capture) that were sequenced in a half lane

^b^L2: Samples (indexed genomic libraries after capture) that were sequenced in a different half lane

^c^Sh: samples sequenced using shotgun approach

#### Target capture and sequencing

We performed whole genome capture using custom *B. burgdorferi* probes of 30 unique field-collected infected tick samples. These samples were previously genotyped and include 14 MLST sequence types and 9 IGS types (Additional file 1: Table S1). The spirochete load for all the analyzed samples, measured by qPCR, fell within the expected range of field-collected nymphs (median = 784 genome equivalents, range 157–3156 copies) [36] (Table 1). Sample DNA content was a conservative estimate of the range of DNA yield within field-collected nymphal ticks extracted through standard procedures (mean = 111 ng, range, 53–189 ng) (Table 1).

The hybridization capture approach effectively enriched for *B. burgdorferi* in all examined genomic samples. The thirty-five enriched samples (30 field tick samples and five replicates used in the different multiplex schemes, see Methods section and Additional file 1: Figure S1) were pooled and sequenced on one Illumina HiSeq 2500 lane, generating 9.7 Gigabases (Gb) of sequence data. After PCR and optical duplicates were removed, we obtained an average of 276.6 Megabases (Mb) of read data/sample (range: 34.2-2066 Mb), of which a mean of 175.1 Mb (15.2–859.1 Mb) mapped to the *B. burgdorferi* B31 reference genome. Mean capture efficiency across samples was 68.6 % (range 9.59–90.58 %) (Table 1, Additional file 1: Figure S2). On average, 62.7 Mb/sample (11.9–1388 Mb) mapped to the *I. scapularis* draft assembly (~10.1 % of all sequence reads; 2.05 %–25.3 %). Whole genome capture efficiency of *B. burgdorferi* was significantly correlated with proportion of tick-derived reads captured (Additional file 1: Figure S3A) (χ2 = 1.21, p < 0.001) and with the *B. burgdorferi* input sample load (Additional file 1: Figure S3B) (χ2 = 0.376, p < 0.001) in univariate models.

### Whole genome coverage

Coverage statistics were calculated for the entire *B. burgdorferi* B31 reference genome (1.52 Mb), including the linear chromosome (911 Kb) and 21 plasmids (5–53 Kb).

#### Shotgun sequencing

Across the two samples, 17.4 % of the *B. burgdorferi* chromosome was covered at an average coverage of 1.3X. Similarly low coverage was obtained for the longest plasmid lp54 (53 Kb), with 23 % of the sequence covered at 1.3X (Table 1).

#### Target capture and sequencing

**Fig. 1:**
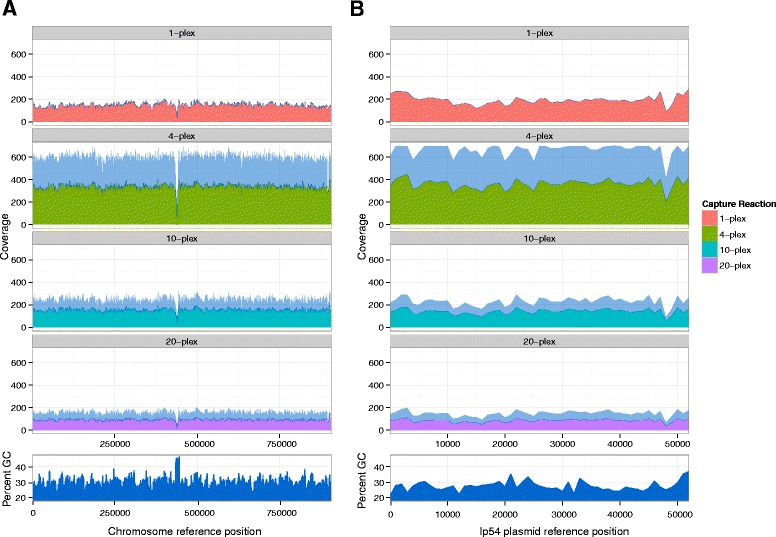
Comparison of the mean coverage for *B. burgdorferi* genomes across capture reactions. **a** The linear chromosome (910,724 bp) and **b** the linear plasmid, lp54 (53,657 bp). The y-axis indicates the coverage depth averaged over all samples in each capture reaction, defined as the total number of sequenced bases which map to each nucleotide in the reference B31 genome calculated on a window size of 1000 bp after removal of potential PCR duplicates and reads with mapping quality ≤20. The x-axis indicates the reference position relative to the samples; the light blue region indicates the area within one standard deviation of the mean coverage of samples captured in the capture reaction. GC content (percentage of nucleotide in the genome that are G or C) for the chromosome and the linear plasmid calculated on a 1000 bp window size are shown below the coverage plots

Across the 30 unique samples and the five replicates, 99.5 % (range: 98.7–99.7 %) of the chromosome was covered at an average coverage of 132X (range: 11.7–597X) (Table 1, Fig. 1a). Two short chromosomal regions between 435–438 Kb and 438–444 Kb exhibited low coverage across all samples (Fig. 1a). These regions contain the 23S rRNA duplicated genes [40]. For the longest plasmid, lp54, the average portion covered was 98.9 % (range: 97.6–100 %) at 133X (range: 12.8–782.6X) (Table 1, Fig. 1b). A lower coverage was observed across the other 9 circular plasmids and the 11 linear plasmids, with 62 % of the plasmid sequences covered at an average of 63-fold coverage, and with 75 % of the plasmid sequences at an average of 80-fold coverage, respectively (Additional file 2: Table S2A and B). Among the circular plasmids, cp32-1 and cp32-8 showed the lowest percentage bases covered (on average 20 % of covered bases at 27-fold coverage and 28 % at 37-fold coverage) (Additional file 2: Table S2A), and among the linear plasmids, lp5 was the only linear sequence showing less than 50 % of covered bases, at an average of 45-fold coverage (Additional file 2: Table S2B).

### Comparison of multiplexing schemes

We assessed the efficiency of capturing *B. burgdorferi* when multiplexing different numbers of samples for hybrid capture reaction. The multiplexing strategy is schematically depicted in Additional file 1: Figure S1. Additional file 1: Figure S2 shows the results of the hybrid capture by multiplexing 1, 4, 10 and 20 indexed tick genomic DNA libraries in a single pool. Table 1 summarizes *B. burgdorferi* capture efficiency for each sample by capture reaction. High average capture efficiency for the 4-plex, 10-plex, and 20-plex captures was consistently achieved (median: 70.3 %, 78.2 %, and 71.5 %), whereas the 1-plex capture sample efficiency was low (10 %). The logistic regression models applied to correlate capture efficiency with the proportion of tick-derived reads and *B. burgdorferi* input sample load, identified the sample captured in 1-plex as a significant outlier (see Methods). We speculate that the 1-plex sample presented low efficiency due to the possible saturation of capture probes by non-specific DNA template molecules. However, we do not have replicates of the 1-plex capture and the low efficiency may reflect the stochasticity of capture.

Per sample chromosomal coverage plots for the ten samples captured in the 10-plex capture reaction, which illustrate the variation expected in coverage among multiple samples captured together, are depicted in Additional file 1: Figure S4.

Five tick samples were captured in duplicate, allowing for direct comparisons of capture efficiency for the same starting genomic library and pathogen load. Table 2 compares capture efficiency among sample replicates. Although the total number of reads generated per sample was inversely correlated with the number of samples pooled, no significant changes in efficiency between captures was observed, except for the 1-plex capture, a clear outlier with significantly lower efficiency than the other captures (Tables 1 and 2, Additional file 1: Figure S3A, B.)

**Table 2:** Pairwise comparison of multiplex captures. Comparison of B. burgdorferi capture efficiency from tick genomic libraries that were captured twice in different reaction capture using different multiplexing scheme

Sample	No. pooled samples ^a^	Total reads (bp)	*B. burgdorferi* mapped reads (bp) ^b^	*B. burgdorferi* efficiency ^c^	Tick mapped reads (bp) ^d^	Tick efficiency
Bbcap4	1	27,557,241	2,644,289	0.10	6,032,190	0.15
	4	2,514,653	1,399,218	0.56	503,015	0.14
Bbcap2	4	3,169,012	1,939,221	0.61	616,781	0.12
	10	1,445,990	927,938	0.64	281,094	0.15
Bbcap30	4	11,002,588	8,744,400	0.79	582,618	0.03
	10	4,574,392	3,780,942	0.83	244,753	0.04
Bbcap31	10	3,903,612	3,328,957	0.85	269,657	0.05
	20	3,097,722	2,638,052	0.85	199,022	0.05
Bbcap6	10	4,466,415	3,605,584	0.81	260,144	0.04
	20	2,694,537	2,190,349	0.81	136,170	0.04

^a^Number of sample mutliplexed in a single capture reaction

^b^Total number of sequenced bases mapped to the *B. burgdorferi* B31 reference genome after removal of PCR and optical duplicates

^c^Proportion of sequences that mapped to the reference genome after removal of PCR and optical duplicates

^d^Total number of sequenced bases mapped to the *I .scapularis* draft assembly [16, 17] after removal of PCR and optical duplicates

### Genome-wide SNPs and SNP effect analysis

We detected 262,987 nucleotide polymorphic sites for the entire data set, with a range of 437–12,291/genome. An average of 4055 SNPs (range: 49–7126) were randomly distributed across the *B. burgdorferi* B31 chromosome (~1 SNP per 227 bp) (Additional file 1: Figure S5). The program SnpEff v2.0b was used to predict the effect of the identified SNPs on *B. burgdorferi* coding sequence. For this analysis, we focused on the chromosome as plasmids contain large stretches of DNA rearrangements, non-homologous recombinations, and inverted repeats. On average 3670 SNPs were found in coding sequences (CDS), of which 1203 were classified as non-synonymous mutations (Additional file 1: Table S3 and S4). The average ratio of non-synonymous to synonymous (N/S) mutations was 0.49 (not normalized). Pairwise comparison of identified SNPs and number of SNPs within CDS between replicate samples showed consistency in SNP detection and location of SNPs across capture pools (Additional file 1: Table S4).

## Discussion

Our results demonstrate the feasibility of combining hybrid capture techniques with NGS to successfully enrich for a target pathogen, *B. burgdorferi*, directly from field-collected vector samples. Using this approach, we generated high quality genomic resources and a repository of genetic variants for 30 unique *B. burgdorferi* field strains for a variety of downstream applications. We obtained high capture efficiency (66.49 %) and nearly complete coverage of the *B. burgdorferi* genome (99.5 %) at 132-fold coverage across all the 35 analyzed samples. By contrast, shotgun sequencing with no pre-enrichment step was significantly less efficient, resulting in only 0.01 % of *B. burgdorferi*-derived sequences (Table 1). The low efficiency of the shotgun approach is consistent with previous findings using the same approach directly on infected ticks, where on average only 1/3 of *B. burgdorferi* genome/sample was recovered [11].

Obtaining high-quality reads with even coverage of the reference can be difficult for templates with biased nucleotide composition [41]. However, we achieved consistently uniform coverage along the majority of the *B. burgdorferi* chromosome (Fig. 1a), although its nucleotide composition is AT-rich (71 %), suggesting that even low GC content microbial genomes can be efficiently sequenced with this method. The incomplete coverage of some plasmids (Additional file 2: Table S2A and B) is expected as previous studies have found DNA rearrangements and inverted repeats are common across *B. burgdorferi* plasmids [42], rendering mapping difficult with short-read data alone.

Hybrid capture avoids both bacterial culture and whole genome amplification, currently standard methods used to enrich pathogens prior genome sequencing and known to be biased in strain representation and propagation of sequencing errors, respectively [18, 20–22]. Further, our study validates this approach for field-collected samples representative of the *B. burgdorferi* copy number and total arthropod DNA content found in natural vector populations.

In the 30 WGS, we identified high levels of genetic polymorphisms at the chromosomal level (49–7126 SNPs per sample). This is a significant improvement from the coarse resolution provided by the highly-conserved MLST markers [26], previously considered the gold-standard for *B. burgdorferi* molecular typing, and other common markers such as IGS and *OspC* [26, 43].

Comparison of different multiplexed capture pools demonstrates high capture efficiency and genome coverage of *B. burgdorferi* even when multiplexing up to 20 genomic libraries in a single capture reaction (Tables 1 and 2), with equivalent sensitivity in SNP detection observed in the pairwise comparison of the replicates in the 10-plex or 20-plex reaction (Additional file 1: Table S4). Thus, this multiplexed capture method is highly scalable, substantially lowering the capture reaction cost per sample.

Many other VBZ pathogens currently share the same research bottleneck: the difficulty in obtaining a sufficient number of pathogen genomes from suboptimal field samples for population genomic study. Hybrid capture is generalizable and can be used to study both VBZ and other pathogens avoiding the difficulties, biases, and costs inherent to pathogen culture or whole genome amplification. Multiple infections involving multiple parasite species within a single vector specimen as well as mixed infections involving genetically distinct clones of the same parasite species within a single vector are frequent [44]. In the first instance, hybrid capture methods may be applied for population genomic study of several bacterial, protozoan, or viral pathogens co-vectored by the same blacklegged tick vector such as *Anaplasma phagocytophilum, Babesia microti*, and Powassan virus [45–49] using a multiple-pathogen capture array*.* In the second case*,* hybrid capture can enrich for the entire population of a single pathogen infecting a single vector or host. If multiple haplotypes are present within the enriched sample, computational and statistical methods can be used to detect and resolve the composition of mixed infections, a level of pathogen diversity often ignored when using pathogen cultures or multi-locus genotyping with Sanger sequencing [25, 44, 50, 51].

This methodology can also be applied across diverse VBZ systems such as study of African trypanosomes directly from collections of the tsetse fly [52–54] or American trypanosomes directly from the triatomine vector [55]. Finally, we note that the development of a single set of capture probes for a pathogen of interest will enable generation of pathogen WGS directly from vector and host samples (whether human, animal reservoir, or both), enabling study of host specificity and potential transmission bottlenecks.

Additionally, hybrid capture has a variety of other potential applications in public health and molecular ecology for studies in which the target DNA represents 1 % or less of a mixed DNA sample. For example, hybrid capture could be adopted as a non-invasive method for whole genome sequencing of target pathogens directly from fecal samples from human and wildlife species [56–58], archived human and animal samples that have been fixed or frozen, or from environmental samples [59], enabling early identification of known zoonotic pathogen outbreaks and their potential origins.

Hybrid capture methods will allow for highly efficient and massively parallel re-sequencing of genomes of interest for non-model organisms, while minimizing cost and effort of sequencing host DNA. Given the high capture efficiency observed in this study, the average throughput of a HiSeq 2500 lane passing quality control (25.5 Gbp for paired-end 75 bp reads), and length of our target genome (~1.52 Mb), we determine that sequencing of up to 160 whole *B. burgdorferi* genomes to an optimal coverage of 30X can be conservatively conducted on a single Illumina HiSeq2500 lane (requiring only 60 % of a single Illumina lane) and costing approximately $1887 (current in-house cost of a single Illumina HiSeq 2500 lane). The cost of hybrid capture and sequencing for a single pathogen with similar genome size to our study organism, for an equivalent coverage, is approximately $227/per sample when multiplexing at least 10 samples per hybridization capture. This cost estimate includes the costs for the library creation, the synthesis of capture array and the Illumina sequencing (Additional file 1: Table S5). Thus, the efficiency and scalability of the proposed method renders population genomic study of *B. burgdorferi* economically feasible. By contrast, shotgun sequencing of *B. burgdorferi* directly from its vector is highly inefficient, as we obtained only 17.4 % of the pathogen genome at low coverage. To obtain a comparable genome coverage necessary for population genomic study (30X) [60], shotgun sequencing of a single *B. burgdorferi*-infected tick sample would require 75 HiSeq2500 lanes (Additional file 1: Table S5). The equivalent sequencing coverage for a single hybrid capture sample would require a fraction of the shotgun sequencing effort (0.006 % of one shotgun sequencing sample).

We propose that multiplexed hybrid capture can be widely applied to sequence other bacterial, protozoan, and viral genomes that exist in low titers in a variety of mixed DNA samples and will enable fine-resolution population genomic study. Yet, several possible limitations need to be considered. First, a preexisting reference genome for the target pathogen or a closely related species is required for capture array design. However, because probe oligonucleotides can hybridize DNA sequences with up to 78 % nucleotide divergence, probes designed using genomes from closely related species can still efficiently enrich for the target pathogen [31, 61]. Conversely, host reference genomes are not necessary for capture array design. In our study, we took advantage of the available draft genome of the *I. scapularis* tick species to test the efficiency of capture. Second, repetitive elements and recombination events within the target genome may be difficult to resolve. However, our results show that we captured nearly the complete *B. burgdorferi* genome, on average 99.74 % of the chromosome and 99.09 % of the longest plasmid, lp54, even after excluding repetitive elements that could not be resolved by read mapping alone (Table 1**).** Given the nature of the genomic data retrieved, it is impossible to identify recombination events. Exclusion of repetitive elements and detailed mapping of recombination events will not preclude evolutionary studies which focus on the large amount of variation present across the rest of the pathogen genome. Finally, captured variation is limited by the set of probes included in the capture array and the sequence divergence allowed (see above), such that novel gene sequences within the target genome may not be captured. However, previous studies have shown that the rate of gene acquisition in *B. burgdorferi* is higher between than within species, and that this rate is among the lowest in bacterial pathogens [62]. This suggests high genome stability and few lineage-specific genes in *B. burgdorferi,* which reduces the impact of this possible bias in this study system.

## Conclusions

Genomic studies of vector-borne and zoonotic pathogens, such as *B. burgdorferi*, have been hindered by the high ratio of host-to-pathogen DNA in arthropod vectors. We adapted target enrichment methods for study of the tick-borne spirochete *B. burgdorferi* directly from field-derived tick samples. Sequence data enable powerful reconstruction of pathogen transmission chains, phylogenies, and GWAS for cultivable pathogenic microbes. The proposed multiplexed hybrid capture and sequencing method enabled the generation of nearly complete genomes of *B. burgdorferi* and the identification of high density, genome-wide SNPs directly from vector samples. We showed that this methodology is highly scalable and cost-effective and can be applied to a variety of VBZ systems as well as broader applications in molecular ecology.

## Methods

### Sample collection and selection

Field ticks used in this study were collected in 2007 using a standard dragging methodology [63] and preserved in 70 % ethanol. The 30 *B. burgdorferi*-infected host-seeking *I. scapularis* nymphs used in this study were chosen to maximize the geographic and pathogen genetic diversity in Northeastern USA. Samples were collected in eight sites in New York, Connecticut, Massachusetts, and Vermont (Additional file 1: Table S1). The two tick specimens used for whole-tick shotgun sequencing were collected using the same methodology in *B. burgdorferi* endemic sites in Connecticut in June 2012 (Additional file 1: Table S1).

Host-seeking nymphs were identified morphologically as *I. scapularis*, using standard taxonomic keys [64], and confirmed by PCR amplification and DNA sequencing of the 16S ribosomal RNA (rRNA) gene [65] (data not shown).

After DNA extraction from individual ticks [66], quantitation and quality assessment was carried out using the dsDNA HS Assay on a Qubit Fluorometer (Invitrogen) and Agilent Bioanalyzer 2100 instrument, respectively. Each tick sample was tested in duplicate for *B. burgdorferi* infection and load using a TaqMan qPCR assay targeting a 68 bp fragment of the 16S rRNA gene [67].

### Genomic library preparation

Illumina library preparation, hybridization capture, and sequencing was conducted at the Yale Center for Genomic Analysis (YCGA). Library preparation was conducted using a modified Roche/Nimblegen SeqCap EZ Library Short Read protocol [68]. Library concentration was determined using PicoGreen assay (Invitrogen) and size selection was performed on a Caliper LabChip GX instrument (PerkinElmer).

### Capture array design

A custom targeted sequence capture array for *B. burgdorferi* was generated using the Roche NimbleGen SeqCap method (Madison, USA) [31, 69], GC-balanced, biotinylated DNA probes were designed *in silico* to tile 99.7 % of the *B. burgdorferi* B31 reference genome (GenBank: GCF_000008685.2, 1 linear chromosome and 21 plasmids) [14, 15]. This isolate is derived from a tick from Shelter Island, NY, USA, the existing reference most geographically and ecologically representative of our samples. We supplemented this *B. burgdorferi* probe set with probes tiling 128 published IGS haplotypes, and 32 known diverse *ospC* haplotypes [70] because these regions are highly divergent across *B. burgdorderi* strains, and because of the role of *ospC* role in transmission [71–73], in addition to being commonly used as markers in population genetic studies [26, 74, 75]. To remove the probes that hybridized to the tick vector, they were screened against the *Ixodes scapularis* draft assembly IscaW1 (Wikel strain), downloaded from VectorBase [16, 17].

### Multiplexed whole genome capture and sequencing

To optimize the multiplexing strategy for maximum *B. burgdorferi* genome capture efficiency, indexed genomic tick libraries were combined in 1, 4, 10, and 20-plex pools. In addition, five samples were captured twice, in two separate multiplexed pools to compare capture efficiency and SNP detection for the same initial *B. burgdorferi*-load and gDNA library captured in different multiplex reactions (Additional file 1: Figure S1). Equimolar amounts of each indexed genomic tick library were pooled prior to capture for a total of 1 ug total genomic DNA *per* hybridization reaction, according to the multiplexing strategy described above. Samples were heat-denatured and mixed with the custom DNA probes (Roche/NimbleGen) and hybridization performed at 47 °C for 68 h. Samples were then washed with a series of stringent buffers to remove non-specifically bound DNA fragments. The captured fragments were PCR amplified and purified with AMPure XP beads. Sample concentrations were normalized to 2 nM and loaded onto two half lanes of Illumina version 3 flow cells at a concentration that yields 170–200 million passing filter clusters per lane. Samples were sequenced using 75 bp paired end sequencing on an Illumina HiSeq 2500 at Yale Center for Genomic Analysis (YCGA) according to Illumina protocols. Sample de-multiplexing was performed using Illumina’s CASAVA 1.8.2 software suite. Metadata and sequence data of each sample in this study are available in the NCBI under BioProject accession number PRJNA264068.

### Shotgun sequencing

For comparison of genome capture efficiency, DNA shotgun sequencing was conducted on two tick samples: Sh1589 and Sh834 (Additional file 1: Table S1). Sample preparation and genomic library preparation was conducted as described without the hybrid capture step. Genomic libraries were prepared from the two whole tick DNA extracts and sequenced directly on one Illumina HiSeq 2000 lane. The mapping pipeline was identical for both shotgun and captured samples.

### Read mapping, genome-wide SNP detection and SNP effect analysis

Raw Illumina sequence reads for each sample were aligned to the *B. burgdorferi* reference genome (strain B31; [14, 15]) using BWA (v. 0.7.7) [76] with soft clipping of bases with Phred quality score below 20. Duplicate sequence reads were marked using the Picard Suite (v. 1.117) MarkDuplicates (http://broadinstitute.github.io/picard/). The percentage of the bacterial reference genome covered (linear chromosome and plasmids), the genome coverage depth and the GC content of the reference were calculated using custom Python scripts. All statistics were generated after removing PCR and optical duplicates and mapped reads with Phred mapping quality ≤20, meaning that any mapped reads that were not uniquely aligned were excluded from the downstream analysis. To evaluate the proportion of reads mapping to the tick vector raw sequence reads were mapped to the *I. scapularis* draft genome assembly [16, 17, 37], using the same pipeline.

SNPs were identified using SAMtools (v. 0.1.19) [77] with the default parameters. SNP filtering was implemented in the vcftools program [78] to include only those variants meeting specific criteria: minimum SNP Phred Quality 30, minimum read depth ≥10, and insertion and deletions were excluded. All filtered homozygous SNPs were annotated and the effect of SNPs on coding sequence was predicted using SnpEff v2.0b [79] using the *B. burgdorferi* B31 genome annotation as a reference.

### Statistical analysis

We used univariate logistic regression to detect correlates of *B. burgdorferi* capture efficiency, the proportion of sequence reads derived from *B. burgdorferi*. We used a quasi-binomial model, allowing for flexibility in the dispersion parameter, and excluded samples identified as regression outliers using DFBETAS, DFFITS, or diagonal elements of the hat matrix. Statistical analysis was completed in R version 3.1.1 [80].

## Additional files

Additional file 1:
**Supplementary Information for Whole genome capture of vector-borne pathogens from mixed DNA samples: a case study of** ***Borrelia burgdorferi.***
Additional file 2: Table S2AB. Mapping statistics for *B. burgdorferi* circular and linear plasmids.

## Abbreviations

Bp: Base pair
qPCR: Quantitative polymerase chain reaction
MLST: Multi-locus sequence typing
WGS: Whole genome sequence
SNP: Single nucleotide polymorphism
GWAS: Genome-wide association studies
VBZ: Vector-borne and zoonotic diseases
