# Supplementary material for: Whole genome capture of vector-borne pathogens from mixed DNA samples: a case study of Borrelia burgdorferi
Source: BMC Genomics. 2015 Jun 6;16(1):434. doi: 10.1186/s12864-015-1634-x (PMC4458057; doi:10.1186/s12864-015-1634-x)
Supplement: Additional file 1: — Supplementary Information for Whole genome capture of vector-borne pathogens from mixed DNA samples: a case study of Borrelia burgdorferi. [file 12864_2015_1634_MOESM1_ESM.pdf]

Supplementary Information for

Whole genome capture of vector-borne pathogens from mixed DNA samples: a case study of Borrelia burgdorferi

Giovanna Carpi [1*§], Katharine S. Walter [1*], Stephen J Bent [2], Anne Gatewood Hoen [3], Maria Diuk-Wasser [1,4], Adalgisa Caccone [1,5]

[1] Yale School of Public Health, Department of Epidemiology of Microbial Diseases, 60 College Street, New Haven, CT 06520, USA

[2] Robinson Research Institute, University of Adelaide, Adelaide, SA 5005, Australia

[3] The Geisel School of Medicine, Dartmouth College, Hanover, NH, 03755, USA.

[4] Department of Ecology, Evolution and Environmental Biology, Columbia University, New York, NY 10027, USA

[5] Department of Ecology and Evolutionary Biology, Yale University, New Haven, CT 06520, USA

*These authors contributed equally to this work

§Corresponding author

SI Appendix includes:

- Tables S1-S5 (Table S2AB is provided separately as additional file in PDF format)
- Figures S1 to S5

Supplementary Tables

Table S1. Sample description. *B. burgdorferi*-infected tick samples (collection years: 2007 and 2012), sampling site, MLST sequence type (ST), IGS (ribosomal RNA intergenic spacer) genotype, DNA yield from whole single ticks, and *B. burgdorferi* pathogen load measured by qPCR.

Sample ID	Site	State	MLST (ST)[c]	IGS type	DNA content (ng)	*B. burgdorferi* copy number
Sh834[a]	Quaddick State Forest	CT	-	-	250.0	3734
Sh1589	Salt Rock State Park	CT	-	-	263.0	1182
Bbcap1[b]	Franklin Delano Roosevelt State Park	NY	3	2	251.2	442
Bbcap2	Franklin Delano Roosevelt State Park	NY	7	-	179.2	3156
Bbcap3	Franklin Delano Roosevelt State Park	NY	7	3	90.9	388
Bbcap4	Franklin Delano Roosevelt State Park	NY	3	2	102.9	603
Bbcap5	Franklin Delano Roosevelt State Park	NY	7	3	189	1472
Bbcap6	Franklin Delano Roosevelt State Park	NY	3	2	153.6	2641
Bbcap7	Franklin Delano Roosevelt State Park	NY	4	2	128.1	599
Bbcap8	Franklin Delano Roosevelt State Park	NY	1	1	96	269
Bbcap9	Franklin Delano Roosevelt State Park	NY	19	9	198.8	1266
Bbcap10	James Baird State Park	NY	7	3	192	2828
Bbcap12	James Baird State Park	NY	3	2	129	1631
Bbcap13	James Baird State Park	NY	3	2	111.6	3030
Bbcap14	James Baird State Park	NY	3	2	169.6	480
Bbcap15	Yale Myers Forest	CT	226	5	144	437
Bbcap16	Yale Myers Forest	CT	8	4	354	1086
Bbcap17	Grand Isle State Park	VT	m[d]	C7	148	2476
Bbcap19	Grafton Lakes State Park	NY	1	1	298.8	356
Bbcap20	Grafton Lakes State Park	NY	14	6	170.8	783
Bbcap21	Moore State Park	MA	16	7	208.6	787
Bbcap22	Moore State Park	MA	1	1	142.8	2387
Bbcap23	Moore State Park	MA	37	8	298.8	582
Bbcap24	Moore State Park	MA	m[d]	E3	213	301
Bbcap25	Moore State Park	MA	19	9	130.8	157
Bbcap26	Hudson Highlands State Park	NY	3	2	174	290
Bbcap27	Mount Riga State Park	CT	3	2	97.2	579
Bbcap28	Mount Riga State Park	CT	18	8	100.8	701
Bbcap29	Lake Gaillard	CT	m[d]	C5	295	2283
Bbcap30	Lake Gaillard	CT	3	2	285.6	3099
Bbcap31	Lake Gaillard	CT	m[d]	C7	275	1800
Bbcap32	Lake Gaillard	CT	40	5	243.6	813

[a] Samples beginning with “Sh” are samples for which shotgun sequencing was conducted on the genomic library generated from whole infected ticks. Collection year: 2012

[b] Samples beginning with “Bbcap” are those captured with custom *B. burgdorferi* probes prior to high-throughput sequencing on a HiSeq2500 lane. Collection year: 2007.

[c] NCBI Accession Numbers: JF419017-JF419118 (each accession number refers to a unique sequence per MLST gene).

[d] m: represents mixed *B. burgdorferi* infections based on the MLST sequence typing—ticks infected with >1 *B. burgdorferi* clone—which could not be resolved with standard PCR-based MLST typing.

Dashes represent samples for which MLST or IGS sequence types could not be successfully determined.

Table S3. SNP detection and annotation for the 30 uniquely sequenced chromosome.

Sample	Reaction [a]	SNPs (Chr) [b]	SNPs in CDS [c]	Non-synonymous SNPs	Synonymous SNPs	Not-normalized ratio (N/S)
Bbcap22	4	49	42	19	23	0.83
Bbcap13	10	4,310	3,899	1,276	2,623	0.49
Bbcap15	10	4,499	4,072	1,300	2,772	0.47
Bbcap19	10	56	49	22	27	0.81
Bbcap29	10	7,126	6,444	2,129	4,315	0.49
Bbcap32	10	4,592	4,139	1,337	2,802	0.48
Bbcap9	10	5,011	4,605	1,535	3,070	0.50
Bbcap1	20	4,036	3,673	1,203	2,470	0.49
Bbcap10	20	2,313	2,067	706	1,361	0.52
Bbcap12	20	4,775	4,303	1,421	2,882	0.49
Bbcap14	20	4,282	3,869	1,262	2,607	0.48
Bbcap16	20	4,507	4,113	1,358	2,755	0.49
Bbcap17	20	6,425	5,809	1,858	3,951	0.47
Bbcap20	20	4,614	4,175	1,337	2,838	0.47
Bbcap21	20	4,760	4,305	1,384	2,921	0.47
Bbcap23	20	4,621	4,174	1,380	2,794	0.49
Bbcap24	20	3,639	3,281	1,076	2,205	0.49
Bbcap25	20	4,956	4,547	1,502	3,045	0.49
Bbcap26	20	4,257	3,852	1,254	2,598	0.48
Bbcap27	20	4,538	4,100	1,339	2,761	0.48
Bbcap28	20	5,452	4,937	1,636	3,301	0.50
Bbcap3	20	2,739	2,465	826	1,639	0.50
Bbcap5	20	2,985	2,691	918	1,773	0.52
Bbcap7	20	4,304	3,893	1,294	2,599	0.50
Bbcap8	20	73	66	17	49	0.35

[a] Capture reaction indicating the number of genomic libraries captured in multiplex.

[b] Total number of retained homozygous SNPs along the linear *B. burgdorferi* chromosome after variant filtering using VCFtools [1] for coverage depth ≥10, Phred Quality call ≥30, and exclusion of INDELs – insertion and deletions).

[c] Number of homozygous SNP variants predicted by SnpEff v2.0b[2] to lie in coding regions excluding nonsense SNPs.

Table S4. Comparison of SNP detection and annotation between the same samples captured in different multiplex strategies. Five tick samples (Bbcap4, Bbcap2, Bbcap30, Bbcap6, and Bbcap31) were captured in duplicate for pairwise comparison of capture efficiency and SNP detection for the same input genomic library and pathogen load captured in different multiplexed pools. SNP detection comparison between listed samples is for the *B. burgdorferi* linear chromosome (NC_001318, 911 Kb).

Sample	Reaction [c]	SNPs (Chr) [d]	SNPs in CDS [e]	Non-synonymous SNPs	Synonymous SNPs	Raw ratio (N/S)
Bbcap4_L1[a]	1	4,274	3,863	1,262	2,601	0.49
Bbcap4_L2[b]	4	4,283	3,868	1,263	2,605	0.48
Bbcap2_L2	4	4,629	4,192	1,401	2,791	0.50
Bbcap2_L1	10	4,395	3,974	1,301	2,673	0.49
Bbcap30_L2	4	4,283	3,866	1,262	2,604	0.48
Bbcap30_L1	10	4,270	3,858	1,256	2,602	0.48
Bbcap6_L1	10	4,280	3,869	1,263	2,606	0.48
Bbcap6_L2	20	4,281	3,871	1,265	2,606	0.49
Bbcap31_L1	10	4,145	3,751	1,223	2,528	0.48
Bbcap31_L2	20	4,164	3,772	1,220	2,552	0.48

[a] L1: Samples (indexed genomic libraries after capture) that were sequenced in a half lane.

[b] L2: Samples (indexed genomic libraries after capture) that were sequenced in a different half lane.

[c] Capture reaction indicating the number of genomic libraries captured in multiplex.

[d] Total number of retained homozygous SNPs along the linear *B. burgdorferi* chromosome after variant filtering using VCFtools [1] for coverage depth ≥10, Phred Quality call ≥30, and exclusion of INDELs – insertion and deletions).

[e] Number of SNP variants predicted by SnpEff v2.0b[2] to lie in coding regions excluding nonsense SNPs.

Table S5. Per genome sequencing cost: comparison between custom hybrid capture and shotgun sequencing in a core sequencing facility.

Sequencing cost is based on 30-fold coverage of the target genome (~1.5 Mb). Based on the capture efficiency of our experiment (~60%), we estimated that 30-fold coverage of a target genome of this genome size would require 120 million reads. The current cost of one 75 bp paired-end HiSeq2500 lane is estimated at $1887 (Yale Center for Genome Analysis) and generates 340 billion reads, thus only a fraction of a lane is needed for sequencing one genome of this size. The cost for the Nimblegen SeqEZ capture array is derived from current listing price of $72,000 for 96 capture reactions.

		Core Sequencing Facility	
		Target Capture (per sample)	Shotgun Sequencing (per sample)
**Sample prep**	*Capture array* [a]	$75.00	0
	Library prep and hybridization (in-house cost)[b]	$144.00	$160.00
**Sequencing**	*30X coverage (in-house cost for HiSeq2500)*	$8.80	$141,525.00 [c]
**Total**		$227.80	$141,685.00

[a] Capture array cost assuming multiplexing 10 samples per capture reaction.

[b] Core facility costs are derived from those advertised at the Yale Center for Genome Analysis (YCGA).

[c] Cost equivalent to 75 HiSeq2500 lanes to obtain 30X coverage for one *B. burgdorferi* complete genome.

Additional costs and genomic resources required upstream of hybrid capture.

Additional costs prior to hybrid capture: Additional preliminary steps and costs required before hybrid capture include field sampling, DNA extractions, and pathogen screening. Since the specific pathogen infection rate will dictate the amount of sampling needed, we only include a general overview of the effort and costs required for a potential pathogen population genomic study. Given a pathogen prevalence of 5-20%, ~200 samples/(population or site) need to be collected and screened to ensure at least 5-20 positive samples/(population or site).

Genomic resources for capture array design: For the design of the capture array for a target pathogen using the Roche NimbleGen SeqCap method (Madison, USA), the genome of the target pathogen species or a closely related species is required. For culturable microbial species for which the genome are not yet available, the lack of a genome reference is not an impediment.

Whole genome sequencing of pure isolates followed by de novo genome assembly will generate the genomic resources necessary. For capture arrays intended to enrich for multiple parasite species within a single sample or vector specimen, the creation of a dual- or multiple-genome pathogen capture array is possible at no additional costs for the array design and synthesis. However, sequencing costs would increase in order to maintain a desired coverage across both pathogen genomes. Considering that the cost of sequencing is only 4% of the total cost of the hybrid capture (Table S5), this would not significantly increase the overall cost and would enable multi-pathogen genomic studies.

Supplementary Figures

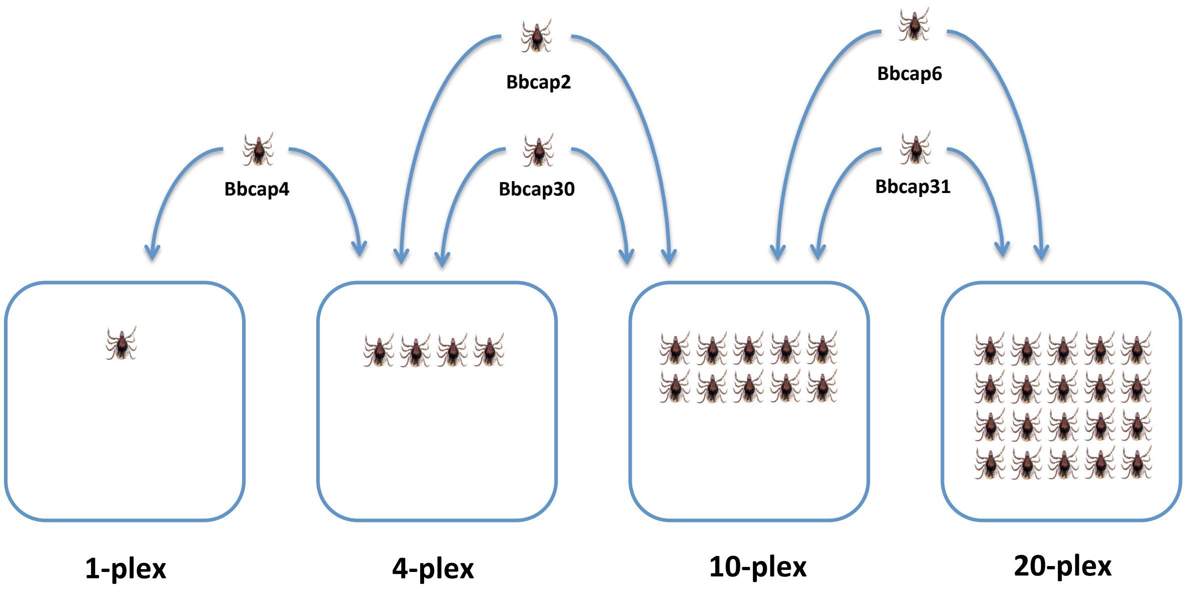

Figure S1. **Multiplexed capture strategy.** A total of 35 tick indexed genomic libraries were captured in four independent reactions in which one, four, ten, and twenty genomic libraries were multiplexed. Capture pools are indicated with blue squares and ticks within each represent the total number of genomic libraries captured in multiplex within that reaction. Five tick samples, represented above the capture pools, (Bbcap4, Bbcap2, Bbcap30, Bbcap6, and Bbcap31) out of 30 tick samples were captured in duplicate, allowing for direct comparisons of capture efficiency for the same starting genomic library and pathogen load. The arrow indicates in which capture pool each tick was used in duplicate. Sequencing and capture statistics for each sample are listed in Table S1.

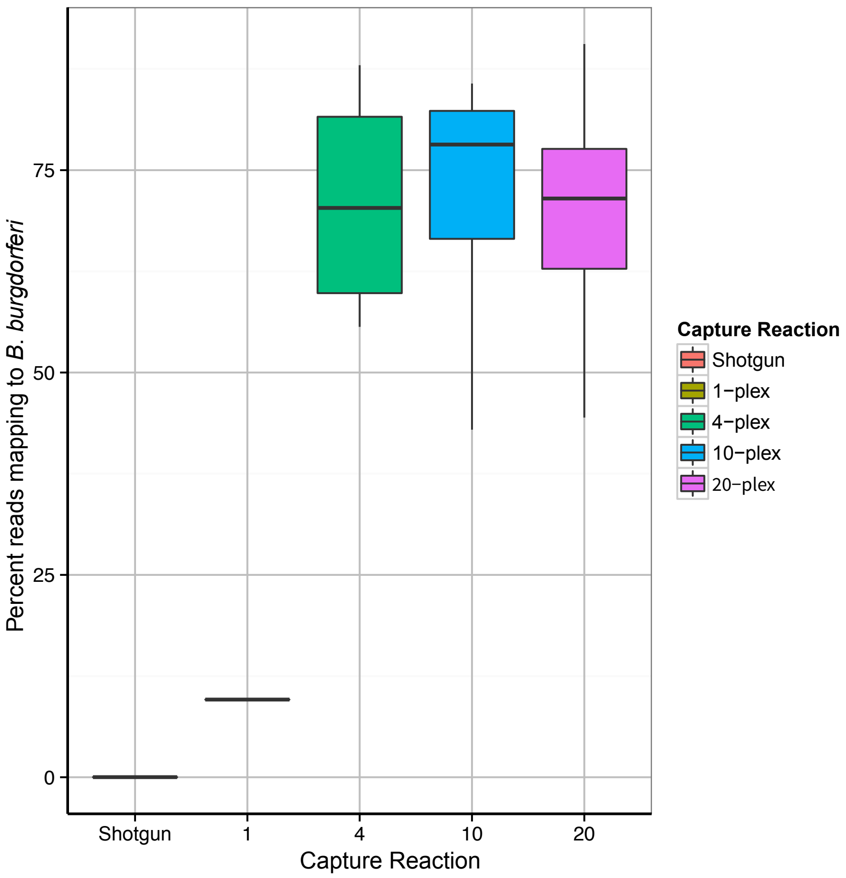

Figure S2. *B. burgdorferi* capture efficiency across capture reactions. The y-axis represents the percentage reads mapping to *B. burgdorferi* reference genome B31 and the x-axis represents the shotgun samples and four capture reactions (1, 4, 10, and 20 multiplexed captures). For each capture reaction, the bold line represents the median capture efficiency, the box represents the interquartile range, and the whiskers represent the range.

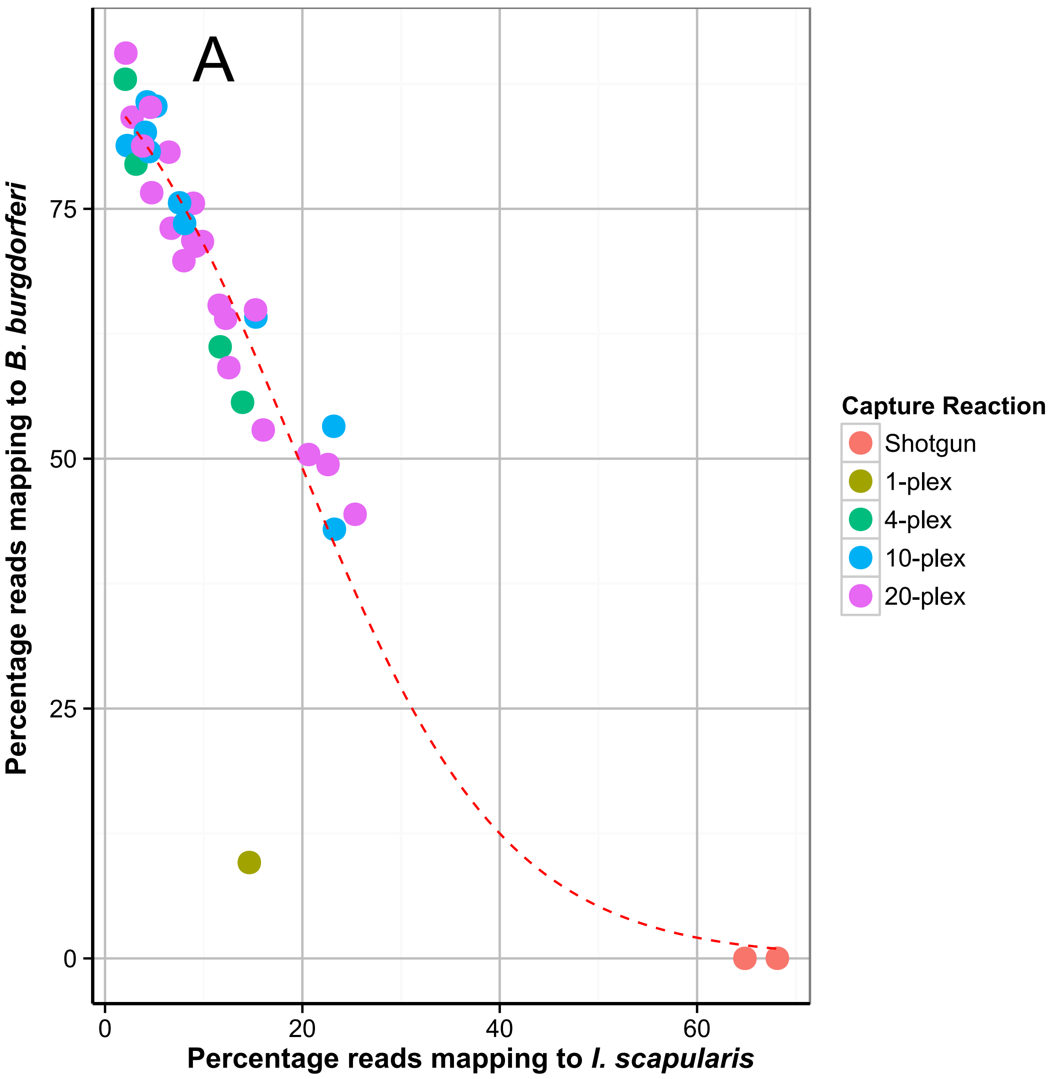
A
Percentage reads mapping to B. burgdorferi
75
50
25
0
0
20
40
60
Percentage reads mapping to I. scapularis
Capture Reaction
Shotgun
1-plex
4-plex
10-plex
20-plex

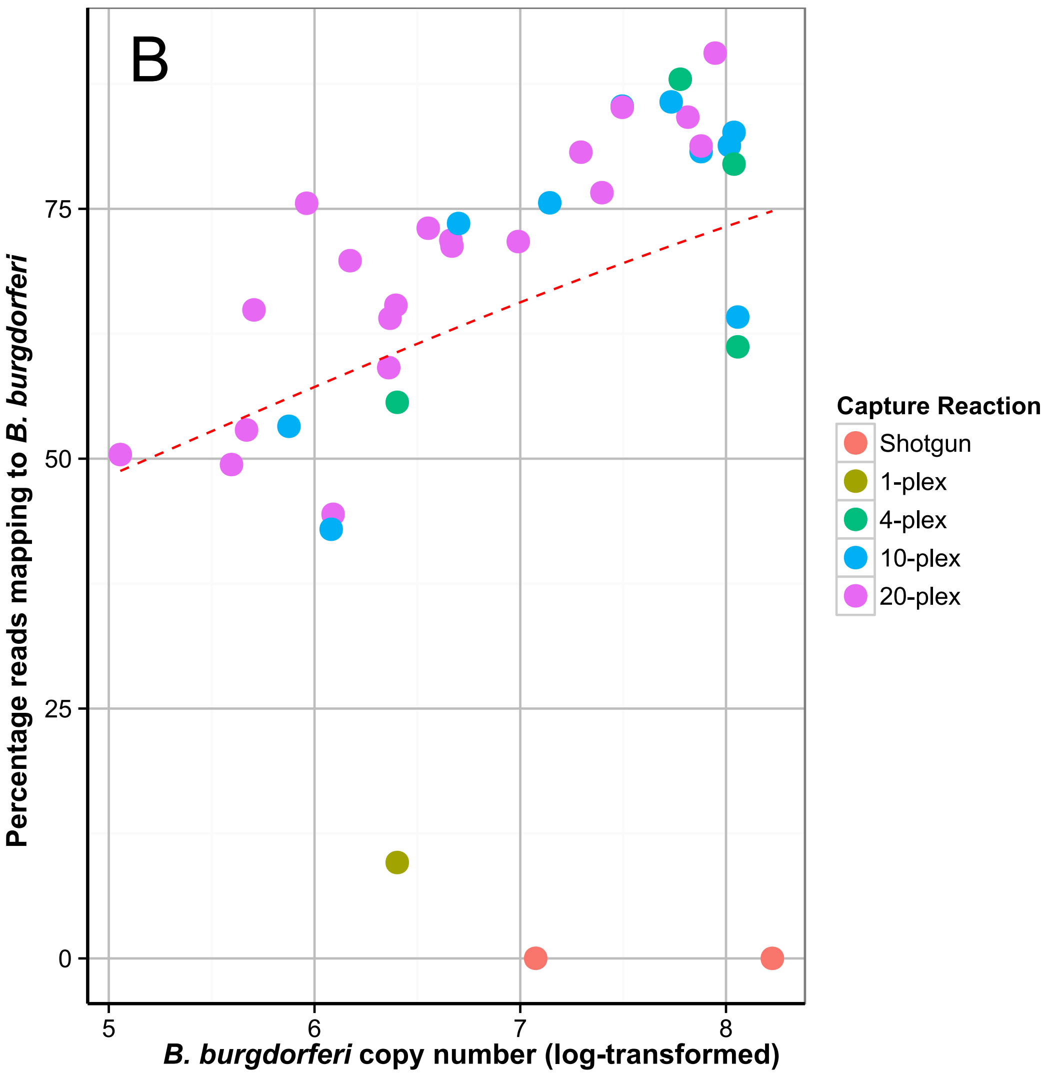

Figure S3. Correlates of *B. burgdorferi* capture efficiency. *B. burgdorferi* capture efficiency is plotted against significant correlates for each sample. Red points denote shotgun samples. Captured samples are represented by dots with different colors representing the reactions in which they were pooled (i.e. green points represent the four samples multiplexed in a single capture reaction). Samples are listed in Table S1. The dashed red lines in each panel represent the best fit logistic regression. (**A**) *B. burgdorferi* capture efficiency is plotted against the proportion of reads mapping to the genome of the vector, *I. scapularis* ($\chi2$ =1.21, p<0.001, 32 degrees of freedom) and (**B**) the *B. burgdorferi* copy number (log scale) or *B. burgdorferi* genome equivalents in the whole tick DNA extract $\chi2$ =0.376, p<0.001, 32 degrees of freedom).

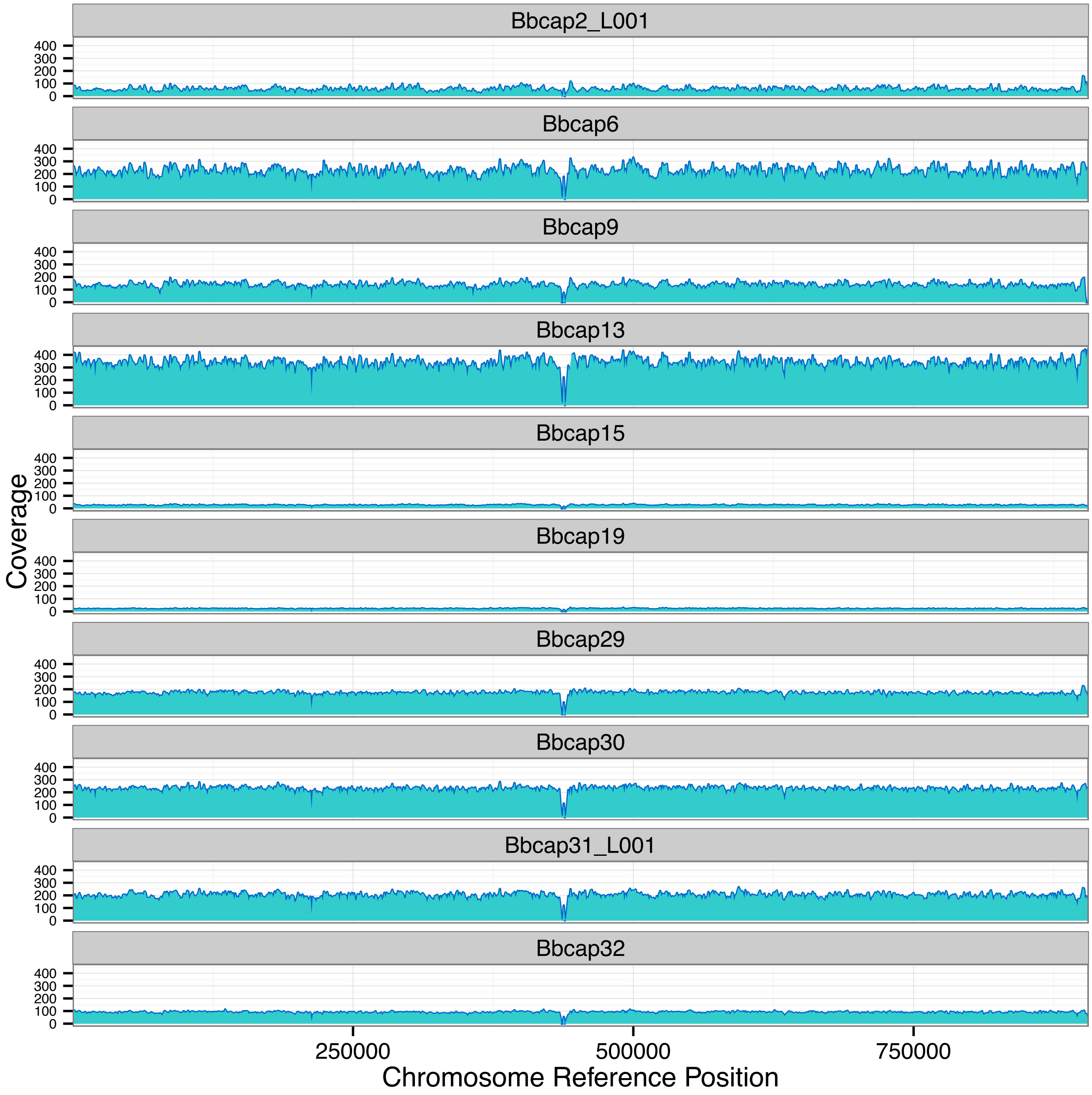

Figure S4. Coverage of the *B. burgdorferi* linear chromosome for each sample captured in the 10-plex capture reaction.

The y-axis indicates the coverage depth of each sample captured in the 10-plex capture reaction. The coverage depth is defined as the total number of sequenced bases which map to each nucleotide in the reference B31 genome after removal of potential PCR duplicates and aligned reads with mapping quality below 20. The x-axis indicates the chromosome position of the reference genome B31. The sample name is indicated above each plot. Two short chromosomal regions between 435-438 Kb and 438-444 Kb exhibited low coverage across all samples. These regions contain the duplicated 23S rRNA genes [36].

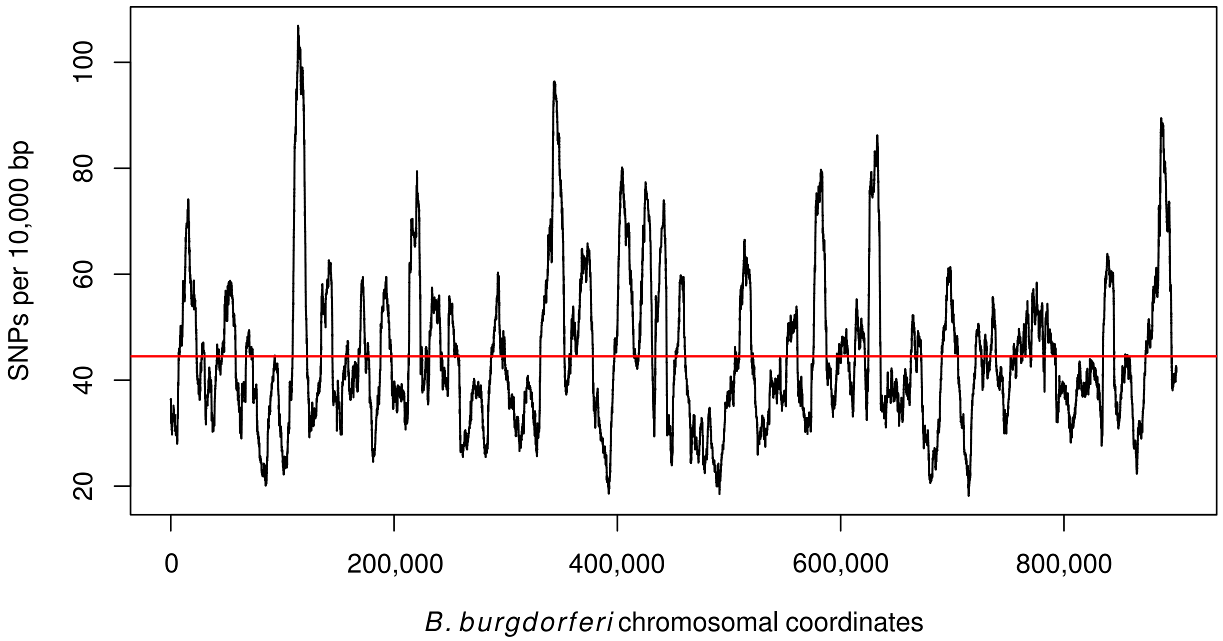

Figure S5. Average SNP density along the *B. burgdorferi* B31 reference chromosome. Chromosomal SNPs in the 35 newly sequenced *B. burgdorferi* genomes were plotted against the reference. The y-axis corresponds to SNP counts per 10,000 bp window; the x-axis corresponds to the *B. burgdorferi* B31 chromosomal position; the red line indicates the average rate of 44 SNPs per 10,000 bp (or 1 SNP per 227 bp).
