# Supplementary material for: Whole genome capture of vector-borne pathogens from mixed DNA samples: a case study of Borrelia burgdorferi
Source: BMC Genomics. 2015 Jun 6;16(1):434. doi: 10.1186/s12864-015-1634-x (PMC4458057; doi:10.1186/s12864-015-1634-x)
Supplement: Additional file 2: Table S2AB. — Mapping statistics for B. burgdorferi circular and linear plasmids. [file 12864_2015_1634_MOESM2_ESM.pdf]

Table S2. Mapping statistics of each sample for the 21 *B. burgdorferi* plasmids.
For each sample, average bases covered and average coverage depth for **A)** circular plasmids and **B)** linear plasmids.

A.

Sample ID	% cp9 Covered	Coverage Deph (cp9)	% cp26 Covered	Coverage Deph (cp26)	% cp32-1 Covered	Coverage Deph (cp32-1)	% cp32-3 Covered	Coverage Deph (cp32-3)	% cp32-4 Covered	Coverage Deph (cp32-4)	% cp32-6 Covered	Coverage Deph (cp32-6)	% cp32-7 Covered	Coverage Deph (cp32-7)	% cp32-8 Covered	Coverage Deph (cp32-8)	% cp32-9 Covered	Coverage Deph (cp32-9)
Bbcap1	16.9	1.4	99.0	126.0	18.7	6.8	40.8	5.9	44.4	4.9	48.1	8.3	53.6	13.1	17.70	4.0	50.1	6.7
Bbcap2_L1	13.0	1.3	100.0	59.8	18.0	5.5	63.0	16.7	79.0	18.3	82.0	26.0	70.0	39.4	33.00	15.3	81.0	19.8
Bbcap2_L2	15.0	1.3	100.0	75.6	21.0	10.4	65.0	33.6	81.0	37.8	84.0	51.9	73.0	77.5	34.00	31.5	83.0	40.8
Bbcap32	81.2	155.9	100.0	30.7	24.6	31.0	54.5	55.7	63.0	31.1	64.7	46.5	81.7	103.6	31.31	27.8	72.2	43.3
Bbcap4_L1	13.6	2.7	99.0	239.3	15.9	41.6	51.9	61.3	59.8	56.5	65.5	83.3	65.9	146.1	33.65	70.2	73.5	59.6
Bbcap4_L2	14.6	1.6	99.0	16.8	16.8	20.5	56.0	29.9	75.2	24.4	69.7	44.9	69.1	77.6	31.47	36.5	79.3	30.9
Bbcap5	98.7	15.8	99.0	92.2	28.4	26.0	64.4	31.5	81.8	27.7	82.8	36.3	74.6	58.7	33.86	26.4	79.5	32.6
Bbcap6_L1	88.3	53.6	99.0	39.7	14.4	38.0	51.3	64.9	59.0	49.0	62.9	84.5	65.2	146.2	33.22	61.8	69.1	63.3
Bbcap6_L2	87.6	32.2	99.0	27.9	15.1	23.5	49.8	40.6	57.6	30.9	63.2	52.6	64.0	91.1	32.40	38.8	67.1	39.8
Bbcap7	97.4	57.2	100.0	11.6	22.0	13.4	44.7	18.9	53.4	10.6	49.1	17.6	64.5	31.6	31.61	13.6	48.6	11.6
Bbcap8	19.3	2.0	99.0	126.1	12.0	3.0	59.4	6.9	86.7	8.9	74.9	10.0	55.7	12.0	16.16	4.2	94.6	9.7
Bbcap9	78.8	183.7	99.0	23.6	15.3	27.9	41.1	42.7	53.2	53.6	50.1	36.6	66.3	79.7	15.26	25.1	53.4	62.8
Bbcap10	18.5	1.6	100.0	21.7	22.2	59.7	67.2	149.0	83.4	116.3	87.0	128.5	74.8	210.7	36.96	106.9	82.3	149.5
Bbcap12	98.9	152.4	100.0	43.3	19.4	27.0	56.4	48.2	66.0	33.4	66.5	65.9	75.7	105.5	34.20	34.7	73.6	43.8
Bbcap13	97.5	1071.2	99.0	60.3	19.5	113.9	55.9	218.6	64.5	148.7	69.4	254.9	71.6	379.5	27.21	96.0	76.8	175.3
Bbcap14	94.8	108.5	100.0	114.0	12.6	17.5	48.9	25.4	55.7	22.7	59.7	31.1	61.8	51.6	30.32	22.5	62.0	27.1
Bbcap15	83.0	51.1	99.0	147.0	10.5	6.5	34.1	10.4	45.8	11.8	44.7	13.4	53.2	21.9	10.95	9.5	42.5	10.2
Bbcap16	70.7	77.1	99.0	29.0	22.5	27.1	41.1	22.1	50.1	17.0	59.8	23.7	56.9	22.2	20.39	18.1	46.0	19.2
Bbcap17	94.1	180.7	99.0	64.3	25.7	49.1	66.3	94.7	82.2	67.3	79.2	105.5	83.1	197.4	36.96	50.7	87.3	71.3
Bbcap19	29.8	2.1	100.0	18.9	6.9	3.8	63.9	16.2	90.7	16.4	76.6	14.3	38.8	10.6	3.22	2.9	96.7	16.3
Bbcap20	74.7	138.3	99.0	307.9	14.3	15.6	61.8	28.4	89.5	26.3	76.0	31.5	70.5	46.8	24.88	10.7	95.5	18.5
Bbcap21	73.6	97.8	100.0	124.0	19.6	29.4	48.9	34.2	61.9	31.2	68.5	39.9	68.3	46.6	27.75	17.6	66.8	20.6
Bbcap22	32.6	9.4	99.0	67.9	19.2	42.1	71.9	410.6	97.2	384.2	91.1	417.3	75.2	286.8	34.20	216.0	100.0	437.4
Bbcap23	85.5	120.4	100.0	97.2	20.9	30.1	40.6	45.0	66.6	27.6	64.4	33.1	59.4	93.8	17.98	31.4	68.1	41.9
Bbcap24	97.4	74.3	100.0	65.2	23.1	6.4	67.3	16.9	87.0	20.1	85.5	20.5	79.6	30.5	35.13	15.5	87.3	17.4
Bbcap25	76.4	35.1	99.0	37.6	13.2	7.4	34.8	10.4	44.0	12.5	37.3	9.2	58.6	17.1	12.16	7.0	47.1	12.3
Bbcap26	97.4	43.2	99.0	121.2	16.2	8.7	48.7	11.5	52.6	11.5	56.2	17.8	61.7	29.0	28.24	12.4	60.9	13.3
Bbcap27	89.5	10.1	100.0	56.2	22.2	14.7	58.6	12.8	78.0	10.0	70.2	18.0	67.2	31.3	27.93	8.2	86.5	11.8
Bbcap28	85.6	97.4	99.0	57.5	23.8	21.4	56.2	24.6	70.7	19.8	66.6	23.6	69.2	61.6	30.05	14.6	76.8	26.2
Bbcap29	100.0	218.7	98.0	8.6	32.7	46.0	67.9	75.4	85.9	44.2	83.0	64.7	86.4	139.7	38.54	50.4	89.0	50.8
Bbcap3	10.5	1.1	99.0	153.8	20.1	12.2	65.3	26.0	78.6	26.7	82.0	32.2	69.2	50.1	32.53	20.9	81.5	27.2
Bbcap30_L1	95.4	20.6	100.0	625.1	24.1	39.3	57.9	73.4	72.5	52.8	75.0	95.1	74.6	159.6	34.78	79.8	78.9	69.4
Bbcap31_L1	97.4	233.5	99.0	56.5	33.3	56.9	69.2	67.3	93.4	55.6	86.5	58.7	79.5	150.0	36.24	49.9	99.6	53.9
Bbcap31_L2	97.5	184.2	99.0	53.4	33.3	44.9	68.2	55.6	94.1	45.3	84.3	47.4	78.9	120.3	36.19	39.1	99.5	43.1
Bbcap32	81.2	155.9	100.0	30.7	24.6	31.0	54.5	55.7	63.0	31.1	64.7	46.5	81.7	103.6	31.31	27.8	72.2	43.3

[a] L1: Samples (indexed genomic libraries after capture) that were sequenced in a half lane.
[b] L2: Samples (indexed genomic libraries after capture) that were sequenced in a different half lane.

Table S2. Mapping statistics of each sample for the 21 *B. burgdorferi* plasmids.
For each sample, average bases covered and average coverage depth for **A)** circular plasmids and **B)** linear plasmids.

B.

Sample ID	% lp5 Covered	Coverage Deph (lp5)	% lp17 Covered	Coverage Deph (lp17)	% lp28-1 Covered	Coverage Deph (lp28-1)	% lp28-2 Covered	Coverage Deph (lp28-2)	% lp28-3 Covered	Coverage Deph (lp28-3)	% lp28-4 Covered	Coverage Deph (lp28-4)	% lp25 Covered	Coverage Deph (lp25)	% lp36 Covered	Coverage Deph (lp36)	% lp38 Covered	Coverage Deph (lp38)	% lp56 Covered	Coverage Deph (lp56)
Bbcap1	37.0	2.7	76.8	13.5	53.2	5.3	39.9	6.7	62.7	16.1	89.7	5.19	86.4	13.7	57.8	8.65	16.0	2.62	25.0	8.02
Bbcap2_L1	30.3	9.7	82.3	29.5	99.7	15.8	84.4	68.9	100.0	46.3	99.3	31.36	97.2	35.1	98.2	32.52	96.5	16.23	32.1	11.21
Bbcap2_L2	29.1	21.4	84.0	63.9	100.0	37.1	86.9	141.0	100.3	100.9	99.7	69.61	98.2	75.1	98.9	71.05	97.9	37.50	37.6	20.10
Bbcap32	70.0	11.2	84.0	56.2	100.0	17.1	83.0	49.6	100.0	51.1	98.0	24.52	96.0	47.2	98.0	31.06	96.0	16.83	31.0	12.31
Bbcap4_L1	20.0	12.7	79.0	116.7	67.0	74.5	48.0	84.5	66.0	233.1	96.0	97.53	91.0	198.2	62.0	169.21	24.0	21.00	40.0	61.54
Bbcap4_L2	14.0	7.0	85.0	53.4	80.0	28.2	77.0	28.5	90.0	95.0	98.0	44.78	94.0	94.7	83.0	64.13	53.0	5.81	53.0	26.85
Bbcap5	41.0	20.9	85.0	51.9	100.0	31.8	89.0	63.2	100.0	84.5	99.0	39.72	96.0	58.6	99.0	51.28	98.0	31.11	37.0	11.39
Bbcap6_L1	46.0	43.9	79.0	159.7	67.0	69.0	39.0	100.5	65.0	306.2	96.0	140.67	91.0	185.5	60.0	143.19	23.0	28.75	38.0	65.13
Bbcap6_L2	47.0	25.9	78.0	96.2	69.0	40.8	48.0	48.8	69.0	177.8	97.0	84.82	91.0	113.3	62.0	83.98	26.0	15.26	36.0	42.50
Bbcap7	85.0	75.1	80.0	42.8	65.0	12.6	54.0	13.0	70.0	48.7	96.0	13.60	90.0	34.6	69.0	26.02	22.0	2.54	32.0	17.24
Bbcap8	15.0	1.2	93.0	20.5	96.0	5.7	98.0	20.0	99.0	14.1	98.0	7.50	96.0	15.2	98.0	9.66	96.0	6.71	82.0	9.31
Bbcap9	90.0	394.4	87.0	75.2	96.0	79.0	9.0	57.8	98.0	115.5	80.0	60.17	87.0	131.8	73.0	72.48	94.0	38.68	36.0	54.02
Bbcap10	29.4	95.5	83.3	518.0	100.3	157.1	89.2	301.5	100.0	291.4	97.1	160.16	97.9	301.8	99.6	279.08	98.8	120.60	34.7	46.11
Bbcap12	79.0	26.1	80.6	126.5	69.2	32.4	53.6	40.1	75.9	178.2	99.6	62.94	92.1	111.0	71.0	65.20	26.8	10.54	72.3	29.54
Bbcap13	26.8	137.7	78.5	656.7	70.3	147.5	46.6	222.2	68.1	607.0	96.8	291.42	93.3	458.6	61.6	368.71	26.6	54.51	42.9	185.80
Bbcap14	42.3	16.8	77.0	108.6	66.7	23.7	49.7	37.5	66.9	98.3	96.3	35.84	90.0	87.3	61.5	52.69	24.3	10.30	35.9	25.57
Bbcap15	45.0	2.2	79.0	23.0	62.5	10.1	26.4	22.5	82.9	14.8	94.7	12.42	86.6	23.3	68.9	17.42	19.0	4.76	27.2	14.05
Bbcap16	41.3	25.6	83.4	55.5	64.4	17.6	46.3	36.8	77.1	57.6	90.0	33.37	92.6	47.5	63.0	48.18	96.4	27.62	33.1	24.84
Bbcap17	32.2	46.9	86.0	197.0	99.4	57.9	89.4	49.3	100.0	184.0	99.9	109.71	96.9	173.5	97.8	87.97	94.8	12.05	47.0	82.18
Bbcap19	n..a	n.a.	94.1	34.8	99.3	8.1	98.7	19.5	100.2	17.9	98.2	8.84	96.7	20.2	98.7	13.60	95.4	6.35	87.3	13.75
Bbcap20	5.3	11.5	91.5	81.0	96.9	17.8	97.2	17.8	99.4	19.9	99.1	29.91	96.2	72.5	97.2	41.71	90.0	5.51	80.7	19.09
Bbcap21	19.4	6.0	80.5	101.8	94.3	22.4	36.7	23.1	85.9	112.4	87.0	44.39	96.0	77.0	69.7	53.18	97.5	19.62	48.3	39.09
Bbcap22	9.8	4.1	97.2	625.6	100.3	364.6	100.3	389.6	100.3	679.6	100.0	457.47	99.2	613.6	100.2	398.45	99.9	248.68	93.5	292.81
Bbcap23	12.5	5.8	77.4	67.6	70.7	25.9	30.3	27.2	50.5	33.2	98.2	63.70	90.0	69.1	61.2	87.55	14.3	4.61	69.5	42.13
Bbcap24	26.3	16.0	84.4	35.1	99.9	13.2	86.6	30.0	99.9	34.3	98.5	15.00	96.5	24.8	98.4	14.90	71.1	2.39	53.1	7.09
Bbcap25	24.4	2.3	85.8	22.8	93.5	9.3	14.3	8.6	95.8	12.0	79.2	8.05	83.5	29.9	71.9	18.05	88.2	5.46	30.6	12.25
Bbcap26	86.6	67.4	77.3	32.5	63.4	11.3	49.6	15.2	66.7	43.6	96.1	13.59	90.2	33.9	60.7	17.98	20.2	5.49	32.8	14.37
Bbcap27	78.7	22.6	90.7	29.7	81.2	8.2	81.2	9.9	95.2	22.1	97.0	11.87	96.8	30.7	91.6	16.36	60.3	2.67	69.0	10.02
Bbcap28	34.7	4.2	86.6	104.3	92.5	21.3	59.2	9.0	91.2	29.0	98.8	45.16	94.8	63.7	76.2	42.66	77.4	3.37	79.8	26.59
Bbcap29	88.5	122.7	89.2	138.8	100.0	32.6	93.9	40.7	100.3	117.4	99.6	83.75	98.2	152.0	99.5	81.73	98.1	14.96	55.9	37.65
Bbcap3	50.8	30.6	83.3	139.8	81.0	73.1	72.4	60.6	88.2	189.1	98.5	198.78	95.2	186.6	83.1	120.53	55.4	16.83	41.5	77.06
Bbcap30_L1	57.8	64.2	84.4	329.3	94.6	152.4	84.1	124.1	98.2	391.2	99.7	474.89	96.1	440.1	96.5	244.30	83.6	26.65	45.7	158.17
Bbcap31_L1	55.7	13.4	96.9	156.7	100.0	69.0	100.2	89.0	100.0	138.2	100.0	76.26	98.0	117.2	100.1	77.05	99.4	47.35	92.1	41.76
Bbcap31_L2	47.1	11.3	96.1	124.5	100.0	53.8	99.9	69.5	100.2	107.8	100.0	60.77	97.7	92.4	99.8	61.26	99.5	37.22	92.3	32.97
Bbcap32	92.3	198.3	80.6	121.2	68.6	35.7	31.5	68.3	71.9	137.7	94.1	44.74	90.6	92.2	65.4	117.88	14.7	7.35	38.4	32.42

Missing value are indicated by n.a. (not available).

[a] L1: Samples (indexed genomic libraries after capture) that were sequenced in a half lane.
[b] L2: Samples (indexed genomic libraries after capture) that were sequenced in a different half lane.
